# Applications of Self-Organizing Maps for Ecomorphological Investigations through Early Ontogeny of Fish

**DOI:** 10.1371/journal.pone.0086646

**Published:** 2014-01-23

**Authors:** Tommaso Russo, Michele Scardi, Stefano Cataudella

**Affiliations:** Laboratory of Experimental Ecology and Aquaculture, Department of Biology, “Tor Vergata” University of Rome, Rome, Italy; University of Zurich, Switzerland

## Abstract

We propose a new graphical approach to the analysis of multi-temporal morphological and ecological data concerning the life history of fish, which can typically serves models in ecomorphological investigations because they often undergo significant ontogenetic changes. These changes can be very complex and difficult to describe, so that visualization, abstraction and interpretation of the underlying relationships are often impeded. Therefore, classic ecomorphological analyses of covariation between morphology and ecology, performed by means of multivariate techniques, may result in non-exhaustive models. The Self Organizing map (SOM) is a new, effective approach for pursuing this aim. In this paper, lateral outlines of larval stages of gilthead sea bream (*Sparus aurata*) and dusky grouper (*Epinephelus marginatus*) were recorded and broken down using by means of Elliptic Fourier Analysis (EFA). Gut contents of the same specimens were also collected and analyzed. Then, shape and trophic habits data were examined by SOM, which allows both a powerful visualization of shape changes and an easy comparison with trophic habit data, via their superimposition onto the trained SOM. Thus, the SOM provides a direct visual approach for matching morphological and ecological changes during fish ontogenesis. This method could be used as a tool to extract and investigate relationships between shape and other sinecological or environmental variables, which cannot be taken into account simultaneously using conventional statistical methods.

## Introduction

Ecomorphology is a comparative discipline that investigates the relationship between shape and ecological features of organisms, species or communities [1]. The basic concept of these researches is that inter-individual morphological variation leads to a different use of the environment and resources [1]–[3]. The investigation of changes in ecomorphological features through ontogeny is an essential topic in this framework, although it has only been partly explored [4]–[11]. Fish are excellent subjects for these studies, since they often undergo through very complex processes of morphogenesis and differentiation during growth. Although the relationship between ontogenetic diet shifts and morphology has been extensively studied [4], [12], there is no clear consensus on whether these two features are related in fish [13]–[15]. In general, the role of changing morphology in inducing ontogenetic niche shifts is not always clear [16]–[22]. The basic step in this field is the identification of patterns in morphology and their correlation with patterns in ecology [2]–[3]; [23] and, consequently, scientific advances are strongly linked to improvements in investigative tools [24]. This is particularly true for morphological studies, since it may be difficult or even impossible to appreciate the ecomorphological aspects underlying biological processes without an adequate shape analysis approach [2]. The modern approach to the description of shape is represented by two alternative (but not mutually exclusive) methods: (1) the homologous point approach, and (2) the boundary outline approach. The former is represented by the Geometric Analysis of landmark coordinates (also called Geometric Morphometry - GM), by means of which information on the spatial localization of morphological variation (its magnitude, position and spatial extent on the organism) can be extracted and communicated. However, GM cannot be used when the number of utilizable landmarks that could be used is low (e.g. when fish larvae are studied), since it leads to non-exhaustive and balanced descriptions of shape [25]. The latter approach is primarily represented by Elliptic Fourier Analysis (EFA) of outline, which allows changes in body profile through the entire ontogeny to be analysed. This approach does not suffer from changes in the number of homologous points arising from organogenesis of the external structure (e.g. fins) [24]. It should be stressed that it is possible to combine these two approaches into the recently developed methodology of semi landmarks [25]–[26], which is based on the detection and analysis of equally spaced landmarks along a curve joining two reference structures or surrounding a body part. However, this approach is aimed at analysing curves in the same analytical framework as landmarks and does not offer advantages with respect to simple EFA if the object of the investigation is the overall shape of the organisms instead of those of single structures or body regions [25]; [26]. Applications of this approach to the study of relationships between shape and ecological characteristics are extensively reported in the literature [7]; [27]–[30].

The classic approach in ecomorphological studies involves the analysis of covariation between morphology and ecology (e.g. shape versus diet, with the latter represented, for instance, as quantitative/qualitative composition of gut contents). In these investigations, the proxies used to describe morphology are generally forced to be very simple (usually confined to linear measurements of external features) in order to be analysed by means of multivariate approaches (e.g. Principal Component Analysis – PCA [31]). Another drawback is that morphology and feeding ecology are non-homogeneous aspects of species sinecology: a common method to measure or describe them and naturally investigate the relations between them does not exist [32]. Moreover, while multivariate statistics can be useful and are familiar to users, they only provide graphical outputs (ordinations, histograms), which must be interpreted. This precludes a direct visualization, abstraction and interpretation of the complex relationships in the original data, especially when the description of shape is involved. Nowadays, researchers in ecomorphology need to visually represent changes in both shape and ecological features during development in order to reveal patterns underpinning data variability in an intuitive and natural way. A new approach for pursuing these aims could be based on a particular type of artificial neural network, known as Kohonen’s Self-Organising Maps (SOMs). This method has already proven useful in pattern recognition and classification [33]–[36]. Furthermore, SOMs are suitable for the analysis of ecological and biological data that are often non-linear, complex, and characterized by internal redundancy or noise [37]. They allow the characteristic patterns of continuous and dynamic processes in complex datasets containing high temporal variability to be identified, affording the visualization of a meaningful two-dimensional model of the input dataset and of the superimposition of all other variables [37]. In recent ecological studies, SOMs were effectively applied to detect the variations existing within genomes spanning the continuum of trophic strategies and then to predict the lifestyle from genome data in bacteria [38] and to investigate relationships between fisheries and environmental factors in Large Marine Ecosystem [39].

The present work examines the application of SOMs to the data obtained from a shape survey of two fish species, from the time of hatching to the end of the larval stage, through EFA of outlines. It was aimed: (1) to describe ontogenetic patterns of shape change in two marine fish species (Gilthead seabream *Sparus aurata* L. and Dusky grouper *Epinephelus marginatus* Lowe); (2) to detect any correspondence between shape and diet changes and to infer their ecomorphological implications through growth.

## Materials and Methods

### Specimen Rearing and Sampling

A total of 362 specimens of sea bream and 199 of dusky grouper were used in this study. Samples were euthanatized with a lethal dose of 2-phenoxythanol (0.5 mg/L). Sampling and killing procedures carried out in this study accomplished the Institutional Animal Care and Use Committee (IACUC) guidelines. The authorization of the Ethics Committee of the “Tor Vergata” University of Rome or some other ethical oversight was not required, because sampling was carried out on a commercial hatchery during year 2004. Samples came from two rearing experiments in which larvae originated from artificial spawning eggs of brood stock and were reared following a semi-intensive approach [26], [31], [40]–[41] using large volume tanks (60 m^3^ in volume, diameter 8 m, water height, 1.2 m). Therefore, the tanks represent ecological mesocosms [41] in which natural nursery conditions (hydrodynamics, environment diversity and prey availability) are simulated [42]. This method is suitable for producing “wild-like” specimens in terms of both external and internal morphology [43], and behaviour [44]. The method involves the connection of the rearing tanks to an external natural lagoon, where a natural zooplankton community is present, via a water inlet provided by a mechanical filter of large mesh (50 mm) [9]. This causes a natural build up of wild zooplankton (copepod nauplii, juveniles and adults, bivalve trochophores and polychaete larvae) in the tanks, resulting in a constant availability of natural food which plays an important trophic role, both in terms of energy source and the learning of feeding behaviour. Cultured live food (*Brachionus plicatilis* and *Artemia salina* nauplii) was also supplied. The availability of live food was analysed daily during the experiment, both in terms of quantitative and qualitative (species composition) description. These data are not presented here in detail, but they clearly show that: a) the densities of various preys were always comparable and high enough to be detected by larvae throughout the rearing period, and b) the preference shown by larvae was not affected by food item densities in the rearing tanks [45].

Specimens were collected at different ages. Each specimen was photographed laterally (on the left side) with a digital camera (24-bit true color images, resolution, 1600 ×1200) and the images collected were processed using the TPSDig2.1 software [46], which also allowed to measure each specimen in total length (TL).

### Shape Reconstruction: Elliptic Fourier Analysis

**Figure 1:**
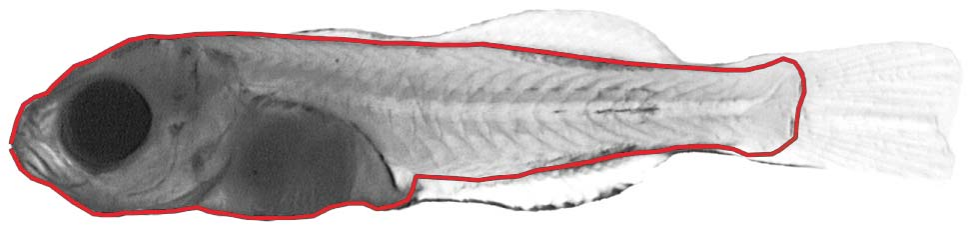
Outline collected on a sea bream specimen.

The outline was collected for each specimen and saved as an array of 50 coordinates using TPSDig (**Fig. 1**), excluding fins, and an Elliptical Fourier function [47] was used to fit specimen outline.

The Fourier series can be expressed as:
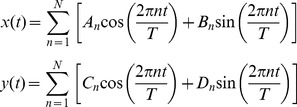
where ***A_1_***, ***C_1_*** to ***A_k_***, ***C_k_*** are known as cosine components, and ***B_1_***, ***D_1_*** to ***B_k_***, ***D_k_*** are known as sine components. The Fourier series can be considered as a series of instructions to deform a basic geometric shape, i.e. a circle, into a more complex one (i.e. the shape of an organism). Each of the successive harmonics, with their four coefficients, represents an additional deformation towards the final shape, which progressively becomes two-, three-, four-, five-lobed, etc. as the number of harmonics increases [48]. As the EFA coefficients do not only depend only on the scale but also on the starting point of the outline, this was taken as the snout tip for all specimens. Before computation, outlines were standardized for size by dividing coordinates by the length of the perimeter of the outline contour.

The number of harmonics required for a precise representation of an outline can be assessed using different criteria. However, if *k* is the number of points sampled, the maximum number of attainable harmonics depends upon *k* and it is equal to either (*k*−l)/2 if *k* is odd or *k*/2 if k is even [46]. In the present study, the optimal number of harmonics was chosen within the set *N* = {5, 10, 15, 20, 25} of five values, using the following procedure [46]: 1) an EFA with *n* harmonics was computed on outlines of specimens of both species, with *n* varying inside *N*; 2) a hierarchical cluster analysis using the Ward’s method was trained on the harmonics coefficients obtained to agglomerate the specimens of both species considered together; 3) the composition of the two groups of specimens separated by the highest node of the cluster analysis was inspected and the number of specimens of each species in each group was counted; 4) the minimal number of harmonics for which these two groups perfectly correspond to the two species (all the dusky grouper specimens in one group and all the sea bream specimens in the other) was retained. In this way, the optimal number of harmonics was found to be 15 and so, given that each harmonic was represented by four coefficients, each fish specimen was described after computation by 60 new variables, generated using the software Morpheus et al. [49].

The matrices thus obtained (one for each species) were used to train two SOMs, one for each species.

### Feeding Ecology of Fish Larvae

The stomach content or, in the first larval stages, the anterior third of the gut was collected and then analyzed. Each food item was identified and counted using a stereomicroscope. Therefore, when approaching the ecomorphological study of the correlation between diet and shape, it was preferred to classify preys not in taxonomical units but by means of artificial, general groupings related to their ecology, habitat, vagility and escape response [15]. This choice was dictated by the fact that, in this work, we were examining the relationships between fish shape and prey type during larval life, and thus the fish size/prey size ratio was a critical aspect. We divided preys into: (1) **small planktonic**, including rotifers, bivalve trocophores and other small, less actively swimming organisms; (2) **medium planktonic**, including copepods, nauplii and juveniles, and smaller ostracods (bosmins); (3) **large planktonic**, including adults of copepods (either calanoids or harpacticoids) and large ostracods (such as Daphnia magna) (4) **mobile benthic**, referring to the organisms living on the surface of the bottom, but not sessile, such as nematodes, crabs and larvae of chironomids.

### The Self-organizing Maps

SOMs were used to display the high-dimensional datasets of fish larvae shape described by EFA in a two- dimensional space, that is a non-linear projection onto a lattice of hexagons. The Kohonen’s self-organizing map consists of two layers: the input layer, connected to each vector of the dataset, and the output layer, consisting of a two-dimensional network of units. The units of each layer are called “neurons”, in analogy with the basic units of the animal neural system. Each unit of the map is associated with a vector of weights, one for each input variable (i.e., in this study, the coefficients of the harmonics). During SOM training, only the input layer is used, so that this procedure is defined as “unsupervised”. The SOM algorithm proceeds by generating a virtual shape unit (VU – the elements of output layer) for each hexagon of the map. The VUs are computed in order to put the sample units (SU - the shape of each specimen which constitutes the input layer) on the map and preserve the neighbourhood, so that similar shapes map close together on the grid. The number of VU is defined by the user and is dependent upon the level of detail desired in the analysis. In this study, the size of the maps was 6×8. The learning procedure is an iterative sequence of steps repeated for a fixed number of epochs/times, measured by the time parameter *t*. The set of weights in the VUs is changed at each iteration of the training phase. The procedure can be summarized in 5 steps:

1) *t* = 0, when the VU_k_ are initialized with random samples drawn from the input dataset; 2) a sample unit SU*_j_*, where *j* varies between 1 and the number of samples, is randomly chosen as an input unit; 3) the distance between SU*_j_* and each VU*_s_*, where *s* varies between 1 and the number of SOM units (48 in this study), is computed using a distance measure; 4) the VU*_c_* closest to the input SU*_j_* is chosen as the best matching unit (BMU); 5) the weights of VU*_c_* and those of the units close to it in the SOM lattice are updated by applying the rule:
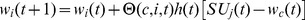
where *w* is the vector of weights for a given SOM unit (in this case w are the coefficients of harmonics), *i* is the index of the SOM unit, *h_t_* is the learning decay coefficient and 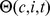 is the neighbourhood function, which returns value depending upon *c* and *i*, that is the relative position of the BMU and of the *i* unit of the SOM; 6) *t* = *t+1* and steps from, 2 to 6 are repeated until *t* = *t_max_*.

The neighbourhood function defines the extension of the VU range of influence, that is how much the change of weights for a given BMU influences the surrounding SOM units and how far this influence propagates on the lattice. In this study, the neighbourhood function was chosen to be Gaussian. Moreover, the learning decay coefficient *h_t_* was chosen to be exponential. The City-Block (Manhattan distance) was chosen as distance measure. The number of training epochs was optimized using the quantization errors (QE) [37]; [50]–[52] as a proxy for learning status. QE represents the mean of the absolute distances between each observation in the training dataset and its BMU in the trained SOM, and it is widely used as a measure of the distortion of the pattern in the SOM: the larger the QE, the worse the obtained SOM pattern. QE decreases monotonically during the SOM training. In this study the learning phase was stopped when the QE reached a minimum and did not show additional decrease when the number of epochs was increased. This empirical approach is usually very reliable, as SOMs do not suffer for over-fitting as other artificial neural approaches (e.g. those based on the back-propagation algorithm). As a consequence, in most cases any reasonably large number of epochs may guarantee a good training and therefore in this study the number of epochs was set to 4,000 for both species.

While it is well know that SOM training is influenced by the initial weights in the neurons, it has been demonstrated that, for non-linear dataset, random initialization represents the best approach [37]. However, the reliability of SOM training was evaluated by performing 100 runs for each species, and computing the QE associated to each run. The SOM with the minimum quantization error was then selected for each species [35].

At the end of the training, the weights of each VU, which corresponded to a series of 60 artificial harmonic coefficients, were transformed back into outlines that can be regarded as “shape prototypes”.

The size (TL) of each fish was passed to the trained SOM as an external variable, and the mean value for each neuron was calculated. If specimens did not occupy the neuron, and then there was no size value, the value being replaced with the mean value of the neighbouring neurons. Finally, size (TL) was visualized on the trained SOMs in a grey-scale. The distances between the adjacent neurons of the maps were visualized by drawing new images of the trained SOMs in which the thickness of the hexagon borders was proportional to the distance between the two neighbouring hexagons. The number of fish specimens assigned to each neuron of the trained SOMs was visualized by means of maps in which a bubble, with the diameters proportional to the number of specimens assigned to that neuron, was drawn for each hexagon. The comparison of the information visualized by the maps with superimposed size, distance-related thickness of the borders and bubble, respectively, was used for the identification of paths in the models representing the shape development for the two fish species. More in detail, the following steps were applied: 1) the two units corresponding to the largest and smallest values of size, respectively, were identified in the trained SOM of each species. These were assumed to be the extremes of the shape path; 2) starting from the unit with the smallest value of size, the adjacent units (neighbours of level one) with values of size larger than the one of the starting unit were identified; 3) among these units, the one with the highest number of assigned specimens was selected; 4) in case of ex-aequo, the inter-units distance (represented by thickness of borders) was considered, and the unit with minimum distance was selected: 5) points 2 to 4 of this procedure were replicated until the units with the largest value of size was reached.

### Trophic Ecology of Fish Larvae

The gut content of each fish specimen was been described by means of the four values of as many binary vectors corresponding to the four groupings reported previously (**small planktonic**, **medium planktonic**, **large planktonic**, and **mobile benthic**). Each vector reports whether any preys of the selected category were found or not in the gut of the specimen considered. These vectors were passed on the trained SOMs, and then the mean value of each food vector in each output neuron was calculated using the size equation reported above for size. Finally, each vector was visualized on the SOM in a grey-scale picture based on the distribution of specimens for each vector. The relationship between shape and food patterns evidenced by SOM was tested using the Mantel test [53]. Distance matrices were computed from both matrices of prototype weights and food (using Manhattan city-block distance), and then compared in order to test the null hypothesis of no-correlation.

## Results

### Self - Organising Maps

It is useful to inspect the result by firstly looking at the SOMs pattern, and then adding the additional information provided by external descriptor (size and diet). SOMs also allow to extract information as the number of specimens assigned to shape prototype, which represents a proxy for the frequency of the observed shape prototypes, and as the largeness of morphological jumps between adjacent prototype, which is a proxy for the differences between morphological stages.

**Figure 2:**
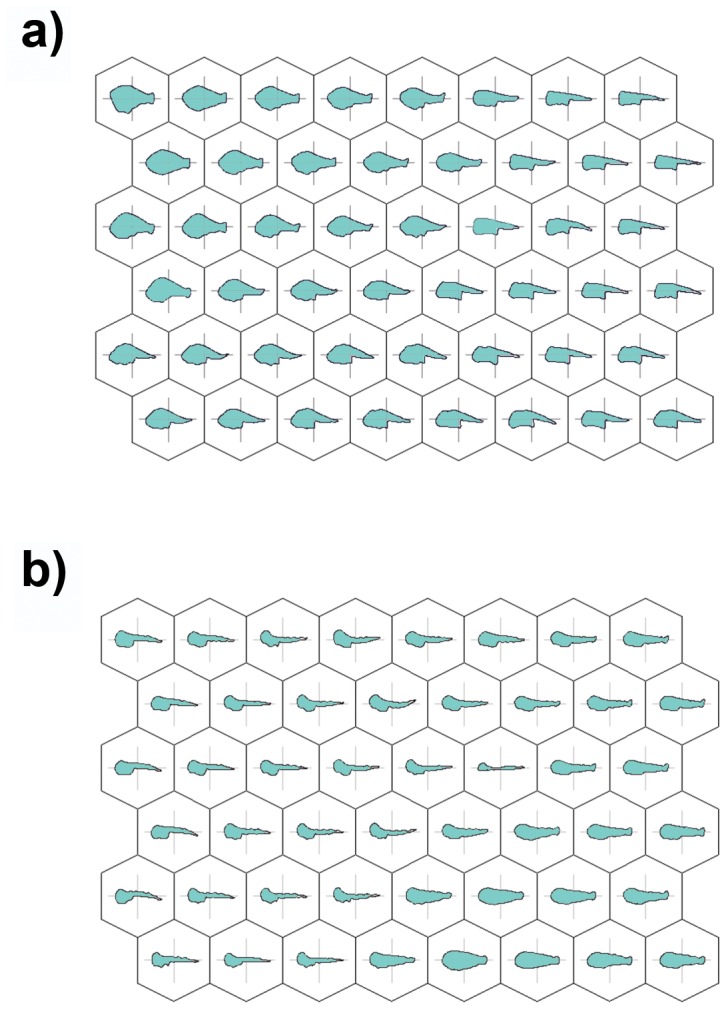
Trained SOM in which virtual shape prototypes are reported for each unit: (a) Dusky grouper and (b) Gilthead sea bream.

The number of SOM output units was 48 (6×8) on a two-dimensional lattice. The trained SOMs classified samples/specimens according to the variation observed in harmonics coefficients. The trained SOMs were reported in **Fig. 2**, showing the virtual fish outlines computed for each SOM unit from weights calculated during training. The outline prototypes represented an abstraction from the information stored in the input data, and their use allowed an effective visualization of shape diversity and variability.

According to these results, the maps point to a set of patterns in shape development: the trajectories in the map that correspond to different ages actually seem to be composed of shapes that have undergone a series of changes. For dusky grouper (**Fig. 2a**), the outlines in the upper right area are characterized by a less developed tail and median region of the body, whereas the head appears to be the largest part. These characteristics are typical of newly hatched and younger larvae. At the bottom of the map, moving from right to left, a series of shape changes involving the tail can be seen, which display relatively positive allometry, while the gut has grown in length and the anus has moved backward. The beginning of the notochord flexion can be detected at the vertical boundary between the third and fourth column of the map. In the upper left area, the outline of a well-developed median region of the body is found, and the growth of this body part is actually the largest shape change detectable in the transition from the lower left side to the upper left one. Therefore, it is possible to observe the initial negative allometry of the head region and the shortness of the tail length, whereas the height of the peduncle increases.

**Figure 3:**
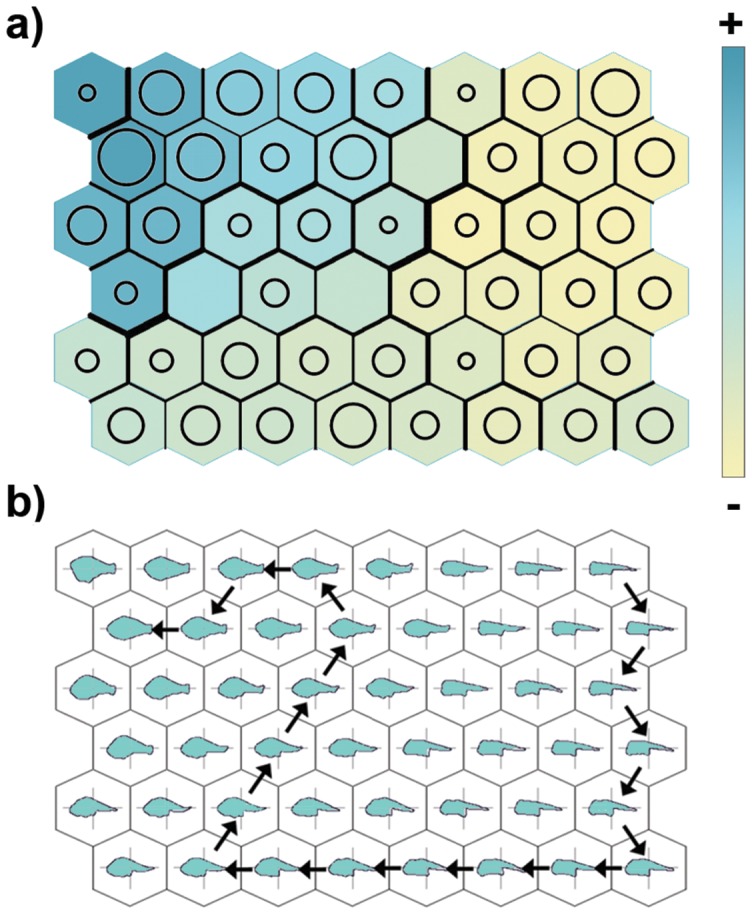
Trained SOM for dusky grouper in which (a) the size (TL) of specimens is visualized by colour scale, while the number of specimens assigned to each map unit is represented by a bubble, and the distance between neighbouring units is represented by differential thickness of the hexagon borders; and (b) a sample path in the shape development is represented by arrows connecting virtual shape prototypes.

Although the sequence started from a different side of the map, a pattern similar to that of sea bream is found in **Fig. 2b**, which presents the output map of the dusky grouper specimens. In this case, the younger specimens are located in the lower left corner of the map, and the subsequent stages are found by moving first right, then up and finally turning to the left. Thus, in this case the trajectory seems to have a left-rotated U shape, and even in this case, a series of empty units separate the extremes of the trajectory. Likewise, the analysis of outline prototypes allowed us, in the bottom left area of the map, to detect outlines with a streamlined body, less-developed median and tail regions and a large head. Moving right, the shape changes begin, especially involving head and tail, which grow more than in the median region of the body. Then, turning left and moving up, it is possible to observe the beginning of the growth of the median region of the body, which becomes progressively higher, while the notochord starts to flex. Starting from the upper right area of the map and moving left, the rearward position of the anus and the growth of the peduncle appear to be the most significant shape change, together with the cessation of notochord flexion. Finally, as we approach the upper left area of the map, it is possible to observe the negative allometry of the head, the increase in height and the shortness of the body and the further strengthening of the peduncle and the tail. These results are further clarified by the visualization of the mean size (TL) corresponding to each unit. **Fig. 3a** shows the map of dusky grouper in which the specimens basically seem to be classified according to their size. It is possible to observe the smallest specimens in the lower right area of the map, whereas the largest specimens are located in the upper left area. It is apparent that different distances exist between different hexagons of the map. In particular, the largest inter-unit distances can be detected in the region connecting the extremes of the shape pattern identified above.

Moreover, the use of the bubble (**Fig. 3a**) indicates that a group of SOM units to which no (or few) specimens were assigned separated these two extremes. It was thus actually possible to detect a trajectory on the map that started from the upper right side, descended and then turned left and finally climbed up to the upper left side. The sample path reported in **Fig. 3b** was obtained by connecting the neighbouring hexagon with the largest bubble, provided that it was associated to a larger fish size (and to the lowest overall distance, in case of ex-aequo hexagons) (**Fig. 3a**).

**Figure 4:**
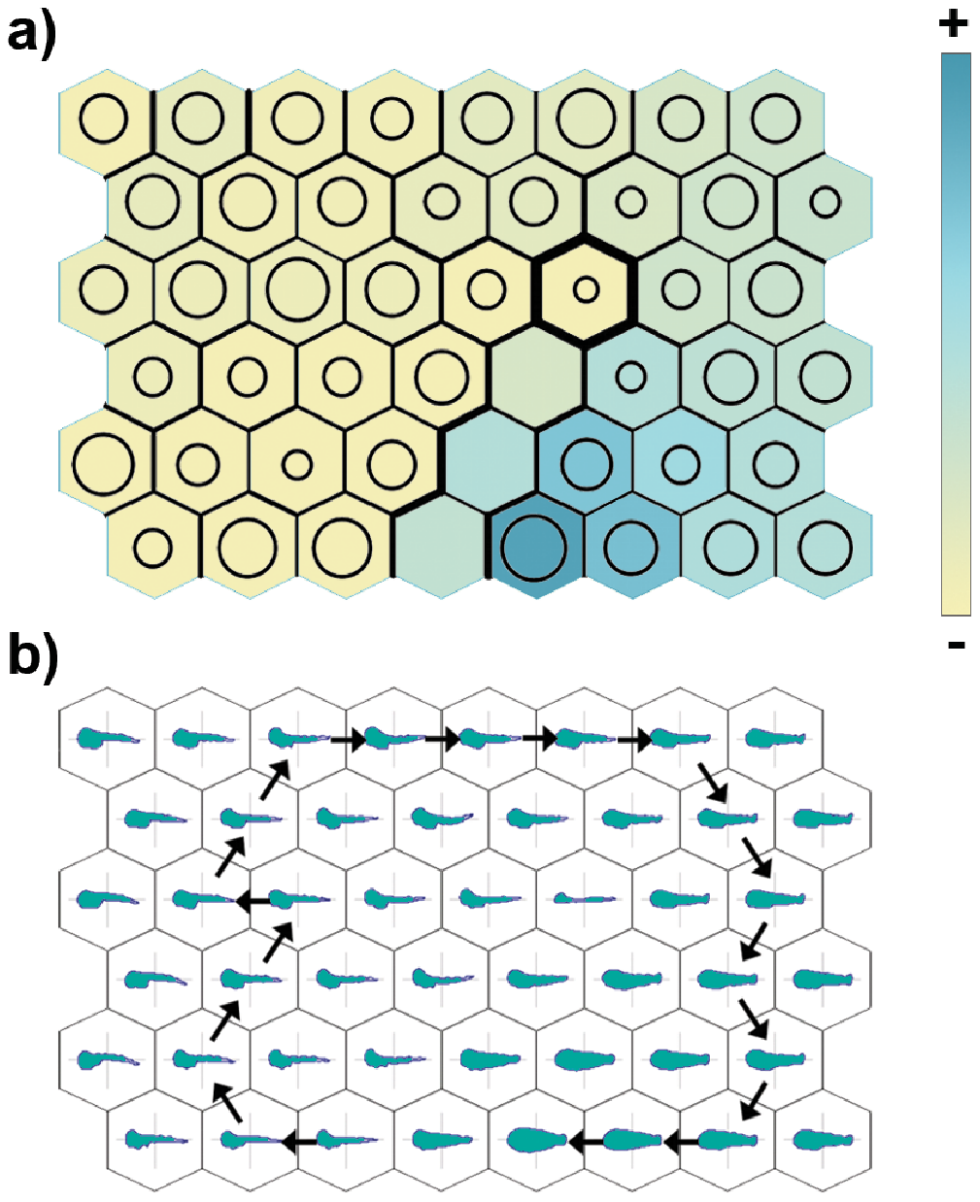
Trained SOM for sea bream in which (a) the size (TL) of specimens is visualized by colour scale, while the number of specimens assigned to each map unit is represented by a bubble, and the distance between neighbouring units is represented by a differential thickness of the hexagon borders; and (b) a sample path in the shape development is represented by arrows connecting virtual shape prototypes.

**Fig. 4** reports the same visualizations for sea bream. Also in this case, the pattern obtained by the superimposition of size (**Fig. 4a**) is in agreement with the pattern of shape changes described above. Likewise, it is possible to identify a region of the map, formed by a linear sequence of hexagons, that is characterized by low value of assigned specimens and a high value of the inter-unit distances (**Fig. 4a**). This “line” marks the boundary between the extremes of the shape pattern modelled by the map. This pattern appears to be circular, as described by the example in **Fig. 4b**.

### Trophic Ecology of Fish Larvae

**Figure 5:**
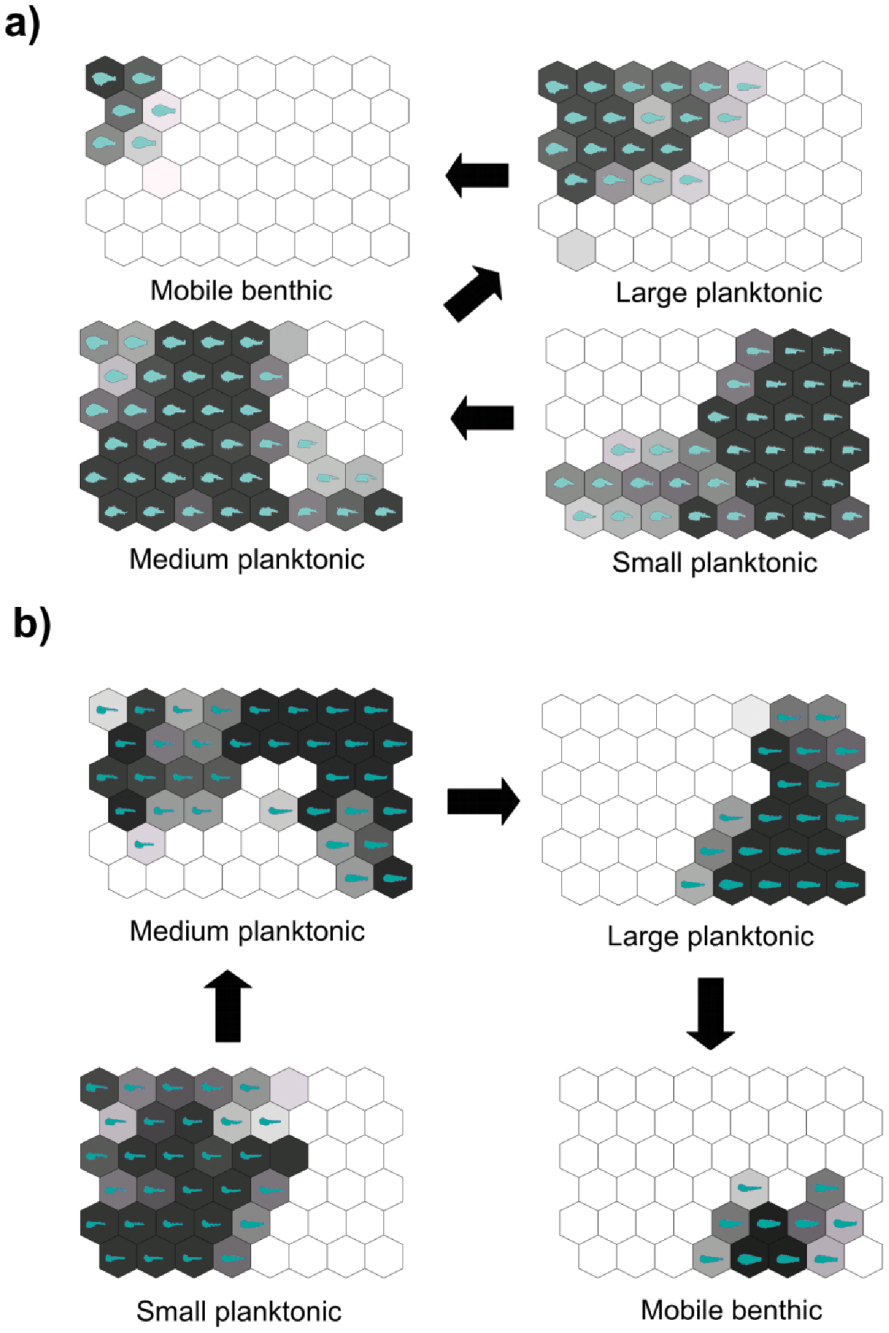
Visualization of the food vectors on the trained SOM maps. The mean value of each vector was calculated in each output neuron of the trained SOMs. Dark grey represents high values while light grey indicated low values. (a) Dusky grouper; (b) Gilthead sea bream. The virtual shape prototypes are also reported for comparative purposes.

Each panel in **Fig. 5** contains the four maps with the superimposition of food vectors. To achieve this, the mean value of each food vector was calculated in each output neuron of the trained SOMs, and then each vector was visualized on the trained SOM map in which the dark area represents a high value, and the light area a low value. These maps show that trophic data for both species match the pattern of shape changes detected by SOMs. The shape prototypes corresponding to different types of food items are also represented to highlight the relationship between shape and diet. It is evident that the four stages into which the development of feeding habit is divided correspond to different regions of the maps, in both cases organized into a succession of hexagon groups.

## Discussion

Self-Organizing Maps are highly efficient in describing ontogenetic trajectories and intra-stage variability, and evidence the major trends in shape changes, which characterize the progressive transition through the different life stages. Furthermore, SOMs detect a series of well-defined shape stages in the ontogenesis of the two species. These stages, detectable as groups of shape prototypes, match the trophic preferences. These findings could well represent a useful contribution to the development of powerful, new tools for ecomorphological investigations.

The study of ontogenetic shape trajectories is a fundamental step in relating the morphological development to the changes in auto-ecological features of organisms, which represents the core of ecomorphology [4]–[8]; [13]; [16]; [17]; [54]; [55]. In this framework, the main difficulties lie in the detection of correspondences between different developmental features (such as shape and trophic ecology) and the assessment of explicit relationships. As evidenced in other studies [37]–[39]; [52], SOMs are one of best tools for analysing biological data when non-linear algebra is involved in the computation or else complex and non-linear relationships characterize the information in the dataset, which could also display noise, redundancy, internal relations and outliers.

For both species studied, SOM training produced maps that organized specimens by size. This allows a time/size sequence of units to be detected in the trained maps. The trajectories obtained for both species were almost U-shaped, i.e. the two ends of each trajectory were relatively close together, although separated by empty units. This result does not imply that the oldest (i.e. largest) larvae are similar in shape to the youngest ones. In fact, SOM units represent virtual shapes and each observed shape was associated with the SOM unit that most closely resembled it. However, distances between SOM units are not a measure of their similarity, because similar shapes are always close together on the SOM, while neighbouring SOM units may be very different [56]–[57]. Basically, a 1-dimensional morphological path certainly explains a very large share of diet variability, but showing how diverse shape can be at different stages and associating each shape to a diet cannot be achieved through a 1-dimensional approach. A SOM can be regarded as an adaptive summary that provides more details (more SOM units) where variability is larger. In fact, two different aspects of shape diversity may be distinguished during growth: diversity among specimens of the same size (or life stage) and diversity among specimens of different size (and thus life stage). The first aspect involves the study of intra-population variability, which provides the basis for the assumption upon which the study of the differential performance of individuals, populations or morphs should be based. The latter aspect represents the “*sensu-stricto*” analysis of developmental changes. It is important to stress that these different aspects lead to two opposite trends in the study: the first approach implies the enhancing of intra-group diversity, whereas the latter aims to ascertain and generalize the developmental patterns. Traditional investigations in ecomorphology are mainly devoted to the understanding of interactions among the different species. Ecomorphology of a single species has only rarely been the focus of a study. Therefore, the simultaneous investigation of ontogenetic development and of intra-specific variability is an unexplored topic.

Considering the ecomorphological evidence regarding this analysis, the observations could be summarized as follows: early larvae of both species have a fusiform body shape, suitable for pelagic life. During growth, the head is the first part of the body to be characterized by positive allometry in both species (**Fig. 3** **–** **4**), which is in agreement with findings for other species [54]; [58]; [59]. This phenomenon may occur because the initial investments in development are allocated to complete the organs most essential for primary functions. Thus, the morphological characters displaying relatively fast growth in early larvae are probably linked to the expected functional priorities for fish larvae, i.e. feeding, gas exchange and swimming [60]–[61]. The changes in head proportions are directly related to the differentiation of new feeding, sensory and respiratory structures [62]. Both the ability to swim and increased activity require well-developed sense organs and a more efficient respiratory system [63].

The central region of the body is the one undergoing major shape changes during the subsequent stages. The growth of gut length and the ventral region, which is possibly associated with the attainment of a discoid shape suitable for manoeuvring [64], seems appropriate prior to switching from planktonic to more benthic feeding habits [54]. The arrangement of shape features in later stages principally involves the acquisition of a discoid body shape. This fact probably reflects an accelerating development of digestive organs, in particular of the intestine [65]. Summarizing the results for both species studied, the patterns of shape changes could be divided into two phases: the first, characterized by the positive allometry of the head and, then, of the tail region and the second by the growth of the median region of the body. The first trend is directly linked to expected priorities of larval development, while the second involves the acquisition of adult shape, and then, of adult auto-ecological features. These findings are consistent with the model of fish ontogeny represented by the theory of saltatory ontogeny. This theory affirms the existence of natural boundaries in the ontogeny of fish, that is changes in structure-to-structure, organ-to-organ and/or organisms-to-environment relationships [63]. The strongest point of this theory is that it affords a better and easier description and interpretation of fish ontogenies, leading to meaningful analyses of general (i.e. interspecific) ontogenetic patterns and of their ecological implications [63].

All vertebrates display a pattern of significantly positive allometry of bite force relative to body and head dimensions during growth [66]. Observing the general pattern of dietary shifts in fish, the transition from small and soft to larger and harder prey is a well-known and common phenomenon. These two combined observations suggest that juveniles do not (or cannot) compensate for their lower absolute levels of performance (e.g. in crushing hard preys), but it seems that the relative performance levels are higher in larger individuals [66]. It could be claimed that small preys are disproportionately abundant in the environment [67] especially if compared to large preys, which therefore remain profitable only for the adult fish that select them. Thus, the existence of intra-specific diet shifts results in a reduction of intra-specific competition for resources by allowing smaller individuals to use different food sources until they reach a size where they can successfully compete with larger individuals of the same species [4]; [7]; [61]; [66].

The findings of this study stress the correspondence between the development of morpho-anatomical features that determine shape changes and changes in dietary preference. Indeed the main advantage of this approach is that it not only allows us to efficiently describe such virtual shapes, and thus the properties of each map unit, but it also enables us to directly link each map unit with a well-defined life-stage condition, which is a step along the ontogenetic trajectory of shape development. This approach results in a very exhaustive analysis of input data pattern, as described in the results, and could be profitably applied to integrate different morphological aspects (e.g. external shape with meristics counts [68]) or external factors such as rearing conditions [69]. However, it should be kept in mind that SOMs are conceived to help users in analysing complex systems by reducing the complexity and point out the main, often non linear, relationship in a qualitative fashion. In this way, SOM are not devised to quantitatively analyse problems (e.g. the relationship between morphometry and diet composition), which could be more properly addressed by statistical approaches (e.g a Mantel test). In the same way, if the researcher is interested in separating well-defined life stages, additional steps can be applied: for instance, clustering the units of the trained SOM allows identifying groups on the lattice [70]. Although many applications of this approach can be found in literature, it is possible to argue that clustering SOM units, which are on their own the output of a clustering-like procedure, may involve elements of circular reasoning.
